# Effects of matching climate change appeals to personal values

**DOI:** 10.1038/s41598-024-56631-z

**Published:** 2024-03-13

**Authors:** Markus Birkenbach, Boris Egloff

**Affiliations:** Department of Psychology, Johannes Gutenberg-University Mainz, Binger Str. 14-16, 55122 Mainz, Germany

**Keywords:** Human behaviour, Psychology and behaviour

## Abstract

The stronger people hold self-enhancing (that is, egoistic or hedonic) values, the less they tend agree with pro-environmental efforts. An exploratory (N = 901) and a confirmatory study (N = 404) examined the effectiveness of pro-environmental messages matched to individuals' values. Findings indicate that strong endorsement of self-transcendent (that is, altruistic or biospheric) values is associated with unspecific endorsement of pro-environmental messages, while individuals endorsing self-enhancement values respond positively only to value-matched appeals.

## Introduction

Climate change poses a threat to both people and ecosystems, and effectively addressing this issue requires substantial efforts by individuals and policymakers^1^. Attempts to persuade individuals about the significance of climate change often involve providing information regarding the associated risks^2^. However, this approach has proven unsuccessful in convincing climate change sceptics of the importance of taking action or to support pro-environmental policies^3,4^. Since agreement with pro-environmental efforts is an important predictor of individual behavior^5^, and perceived public support for pro-environmental policies is an important predictor of the implementation of such policies^6^, researchers have tested alternative communication strategies to increase agreement with pro-environmental efforts. On such strategy is to communicate pro-environmental concerns through the lens of personal values.

Values are defined as guiding principles that shape an individual’s life^7^ and are useful predictors of attitudes, norms, beliefs, and behaviors^8^. Research has identified four values that play a significant role in shaping attitudes toward climate change: altruistic (e.g. benefitting others), biospheric (e.g. appreciating nature), egoistic (e.g. increasing own resources) and hedonic (e.g. doing what feels good)^9^. These four values can be clustered into two broader factors: self-transcendent values (altruistic and biospheric values) and self-enhancement values (hedonic and egoistic values)^10–12^. Higher scores in self-transcendent values are associated with low levels of climate change skepticism^12^, greater engagement in energy-saving behaviors^10,13^ and increased concern and awareness about the environmental consequences of behaviors^14,15^. Conversely, higher ratings in self-enhancement values are either inversely or not at all associated with pro-environmental behaviors, attitudes or intentions: they are linked to climate change skepticism^12^, reduced willingness to reduce motor vehicle usage^16^, self-reports of reduced engagement in energy-saving behaviors^10^ and are not associated with the intention to behave in a pro-environmental manner^8^.

Based on these findings, and considering that exclusively informative interventions tend to only influence individuals who already strongly endorse self-transcendent values^4^, researchers have designed experiments focusing environmental values. Interventions targeting self-transcendent values, such as promoting environmental benefits, generally find that stronger self-transcendent values are associated with increased positive emotions toward environmentally beneficial products^17^, increased measures in some laboratory measures of pro-environmental behavior like taking time to inspect paper saving tips^18^, and information on benefits of car-sharing even spilled over to recycling behavior^19^. On the other hand, interventions targeting self-enhancement values, such as emphasizing financial benefits or visual appeal of a product or behavior, have yielded mixed results: while stronger self-enhancement values appear to be linked to some laboratory measures of pro-environmental behavior^18^, they are not associated with more positive emotions toward a product^17^ and may even have negative effects on behavioral spillover^19^. Mixed appeals that present both types of stimuli in succession generally find weaker effects on liking and pro-environmental behavior compared to purely self-transcendent stimuli^18,19^. Some authors suggest that this could be due to the combination of value-congruent and value-incongruent framing diluting the effect^18^ or that self-enhancement framing of pro-environmental behavior might be ineffective, given that such behavior is inherently self-transcendent in nature^19^. Proposed solutions to this discrepancy include strengthening self-transcendent values but the lack of empirical evidence on the efficacy of such interventions^11^ hinders easy implementation of this proposal. Taken together, these results may lead to pessimism: individuals who strongly endorse self-enhancement values may seem unresponsive to the self-transcendent goals of pro-environmental behaviors, such as acting on climate change.

However prior research has shown that agreement to pro-environmental ideas increases when these are communicated in accordance with personal values. Researchers found that agreement with pro-environmental policies is higher for individuals high in self-enhancement values if these policies are explained by highlighting personal benefits^20^, and university students high in self-enhancement values were more likely to sign up for a beach clean-up event if the personal benefits of the event were highlighted^21^.

Taken together, it is entirely possible for individuals to support pro-environmental efforts for self-interested reasons, such as safeguarding their own well-being and maintaining their standard of living. We were interested to see whether these effects can be replicated and translated to other types of stimuli. With this perspective in mind, we developed four posters containing messages tailored to different values, emphasizing the importance of pro-environmental efforts (e.g., ‘egoistic’: “protecting the planet means: protecting your own well-being” or ‘altruistic’: “protecting the planet means: doing something good for everyone”; see Supplemental Material 1). These posters were presented to a community sample of 901 participants in pre-studies and 404 participants in our preregistered main study using a within-group design.

We first analyze the association between values and agreement with the presented posters using linear regression using self-enhancement and self-transcendent values as predictors and the poster ratings of the posters targeting self-transcendent or self-enhancement values as dependent variables (step 1). To control for overall agreement with pro-environmental messaging and other, unsystematic variances such as response-styles, we included the rating of the other poster in a second step as an additional predictor in the regression model (step 2). In our analysis of the pre-studies, we discovered that individuals who hold stronger self-transcendent values tend to rate all pro-environmental posters more favorably (β between 0.281 and 0.370, all *p* < 0.001; see Table 1). Conversely, individuals with higher scores in self-enhancement values exhibited a positive association only with ratings of posters that focused on self-enhancement values (β = 0.171, *p* < 0.001). However, when we considered overall agreement with pro-environmental messaging (Step 2 of the regression analyses, right half of Table 1), we observed that self-transcendent values were only associated with ratings of posters that specifically targeted self-transcendent values (β = 0.191, *p* < 0.001). Similarly, self-enhancement values remained positively linked to agreement with self-enhancement posters (β = 0.142, *p* < 0.001) but became negative predictors of ratings for posters focusing on self-transcendent values (β = − 0.064, *p* = 0.009).

**Table 1:** Linear regression predicting poster ratings using self-transcendent and self-enhancement values.

	Step 1		Step 2	
	β	*p*	β	*p*
Self-transcendent poster rating				
Pre-study				
Self-transcendent value	0.370	< 0.001	0.191	< 0.001
Self-enhancement value	0.045	0.146	− 0.064	0.009
Other poster-rating			0.635	< 0.001
R^2^	0.14		0.50	
Main study				
Self-transcendent value	0.396	< 0.001	0.282	< 0.001
Self-enhancement value	0.089	0.054	− 0.032	0.378
Other poster-rating			0.619	< 0.001
R^2^	0.18		0.53	
Self-enhancement poster rating				
Pre-study				
Self-transcendent value	0.281	< 0.001	0.039	0.135
Self-enhancement value	0.171	< 0.001	0.142	< 0.001
Other poster-rating			0.654	< 0.001
R^2^	0.12		0.48	
Main study				
Self-transcendent value	0.186	< 0.001	− 0.087	0.031
Self-enhancement value	0.196	< 0.001	0.134	< 0.001
Other poster-rating			0.689	< 0.001
R^2^	0.09		0.48	

Pre-studies: N = 901; main study: N = 404.

In our preregistered main study (available at 10.17605/OSF.IO/8Y346), we were able to largely confirm the findings from the pre-studies. Again, we observed a positive association between self-transcendent values and ratings of posters, regardless of the targeted values (β between 0.186 and 0.396, all *p* < 0.001; see Table 1). On the other hand, self-enhancement values were positively linked only to posters that targeted the corresponding self-enhancement values (β = 0.196, *p* < 0.001). When controlling for overall agreement with pro-environmental messaging, self-transcendent values maintained a positive association with ratings of posters targeting self-transcendence values (β = 0.282, *p* < 0.001), but became negative predictors of posters targeting self-enhancement values (β = − 0.087; *p* = 0.031). Applying the same controls, self-enhancement values were positively associated with ratings of posters targeting self-enhancement values (β = 0.134, *p* < 0.001), but were independent of ratings of posters targeting self-transcendent values (β = − 0.032, *p* = 0.378).

Taken together, the pattern of results observed consistently across the pre-studies and the main study can be summarized as follows: when taking overall agreement into account, there was a specific matching effect for both values, whereby self-transcendent values were associated with higher endorsement of self-transcendent posters, and self-enhancement values were associated with higher endorsement of self-enhancement posters. However, when not controlling for overall agreement, specificity was only evident for self-enhancement values, while self-transcendent values were associated with general endorsement of both types of posters.

Please note that the two findings that differ in terms of statistical significance (*p* < 0.05) between the pre-studies and the main study are (1) of small absolute magnitude (β ranging from − 0.087 to 0.039), and (2) significant primarily due to the large sample size and the chosen significance level. If a more stringent significance level such as *p* < 0.005 were applied (arguably more appropriate^22^), these effects would consistently yield null results. This highlights the justifiability of not interpreting these effects as meaningful.

It is worth noting that, even though we surveyed a relatively large sample and largely replicated our results between the pre-studies and the main study, the cross-sectional and correlational data necessitates further research to explore potential causal effects of personal values. Investigating such causal effects may be challenging, as experimentally manipulating personal values is difficult and may require longitudinal data. However, we believe that the next crucial steps in understanding the significance of personal values and targeted messaging in the discourse on climate change would involve (a) examining the associations between personal values, agreement with pro-environmental appeals, and actual behaviors such as individual engagement (or lack thereof) in pro-environmental actions or support for relevant policies and (b) clarifying potential drawbacks of value-incongruent messaging, for which we found weak and inconsistent evidence in both samples. Our data support the finding that value-congruent messaging might lead to a more positive attitude toward pro-environmental efforts. But attitudes, while an important predictor of behavior, are certainly not sufficient to translate to actual behavior^23^. Interestingly, it has been argued that pro-environmental behavior does not need to be done out of personal conviction, and financial incentives might be a way to increase engagement in pro-environmental behavior without personal conviction^24^. However, since the implementation of financial incentives usually are policy decisions, we believe that a more positive attitude toward pro-environmental efforts has value in itself, since public opinion is relevant for policymakers.

Overall, our findings indicate that aligning pro-environmental appeals with personal values is effective. Individuals who strongly endorse self-transcendent values tend to agree with pro-environmental appeals regardless of the specific content of the posters, whereas those who endorse self-enhancement values may only agree with pro-environmental appeals when they are aligned with their specific values. Our findings may hint that value-incongruent messaging might even lead to more disagreement with pro-environmental efforts, also highlighting the potential negative impact of unspecific (‘one-size-fits-all’) appeals. The reasons for these potential drawback-effects (please note that these drawback-effects only appeared in one of our samples) might be manifold and may include annoyance, heuristic information processing^25^ or derogation of a perceived “moral superiority” and research into sources of disagreement can help understand these effects further. We would like to stress that this effect did not emerge clearly across both pre-studies and main study and requires further research. Given the inconsistent effects, we believe that this potential drawback effect is of small size at most. Importantly, individuals with strong self-enhancement values can likely be convinced to support action on climate change, but this is contingent upon appeals that emphasize self-enhancement reasons for taking action. Thus, it is not contradictory, but rather entirely possible, for individuals to agree with pro-environmental efforts for selfish or self-enhancement reasons, such as safeguarding their own well-being and standard of living.

## Method

All experiments were conducted with participants recruited via Prolific. Completion time was estimated at seven minutes and participants were paid 1.05£ in the pre-studies. Due to improved estimates, completion time was estimated at five minutes and participants were paid 89 ct in the main study. The study design was approved by the ethics committee of the department of psychology at the Johannes Gutenberg-University Mainz. Informed consent was given from all participants and all experiments were performed in accordance with the Declaration of Helsinki. The final sample of the prestudies included 901 participants. 449 (49.8%) were male, 448 (49.7%) female, 3 (0.3%) non-binary and one (0.1%) trans male. Participants were on average 39.20 years old (SD = 13.19). Our main study sample consisted of 404 participants, of which 202 (50%) were male, 200 (49.5%) female and 2 (0.5%) non-binary. After giving informed consent, participants completed the Environmental Portrait-Value-Questionnaire (E-PVQ^10^). They then were shown four posters in a random order and were asked to rate the poster after viewing each poster. The poster background was a picture of the earth from space on a black background. Each poster contained three lines of text in a bold white font (detailed study materials are available from 10.17605/osf.io/6YW8N, posters are shown in Supplemental Material 1). The texts on the posters tailored to self-transcendent values were “Because everyone will benefit—Protecting our planet means: Doing something good for everyone—Save our planet” and “Because nature is important—Protecting our planet means: Preserving nature—Save our planet”. The texts on the posters targeting self-enhancement values were “What’s good for the planet is good for you—Protecting your planet means: Protecting your own well-being—Save your planet” and “Because earth is where the fun is—Protecting our planet means: Doing everything you like in the future—Save your planet”. Rating questions were adapted from a prior article^26^: “I find this poster to be persuasive”, “This is an effective poster”, “Overall, I like this poster” and “I am interested in learning more about saving the planet after seeing this poster” presented with a 7-point Likert scale (1 = very strongly disagree; 7 = very strongly agree). Posters are available in the Supplement. We used the mean value of these four items as the poster-ratings. Descriptives and intercorrelations of all study variables are displayed in Supplementary Tables 1 and 2. Intercorrelations of the rating items are displayed in Supplementary Table 3.

## Analyses

We aggregated the altruistic and biospheric values to a single value (self-transcendent) and then aggregated the egoistic and hedonic value to a single value (self-enhancement). We also averaged the ratings of the altruistic and biospheric poster as well as the ratings of the egoistic and hedonic poster. We first analyze the association between values and agreement with the presented posters using linear regression using self-enhancement and self-transcendent values as predictors and the poster ratings of the posters targeting self-transcendent or self-enhancement values as dependent variables (step 1). In order to control for overall agreement with pro-environmental messaging and other, unsystematic variances such as response-styles, we included the rating of the other poster in a second step as an additional predictor in the regression model (step 2). Detailed results are displayed in Supplementary Tables 4 and 5.

### Supplementary Information

Supplementary Information.

## Data Availability

The data used in this study are available on the project page on the Open Science Foundation under 10.17605/osf.io/6YW8N.
