# Supplementary material for: Effects of matching climate change appeals to personal values
Source: Sci Rep. 2024 Mar 13;14:6128. doi: 10.1038/s41598-024-56631-z (PMC10937651; doi:10.1038/s41598-024-56631-z)
Supplement: Supplementary file 1 — Supplementary Information. [file 41598_2024_56631_MOESM1_ESM.docx]

Supplementary Table 1. Descriptives

|  | Min | Max | M | SD |
| --- | --- | --- | --- | --- |
| pre-studies |  |  |  |  |
| Age | 18 | 80 | 39.20 | 13.19 |
| Value: self-transcendent | 1.33 | 7.00 | 5.60 | 0.85 |
| Value: self-enhancement | 2.00 | 6.75 | 4.52 | 0.80 |
| Poster rating self-transcendent | 1.00 | 7.00 | 4.25 | 1.17 |
| Poster rating self-enhancement | 1.00 | 7.00 | 4.03 | 1.18 |
| Main study |  |  |  |  |
| Age | 18 | 80 | 38.67 | 12.29 |
| Value: self-transcendent | 1.00 | 7.00 | 5.57 | 0.93 |
| Value: self-enhancement | 1.00 | 7.00 | 4.57 | 0.81 |
| Poster rating self-transcendent | 1.00 | 7.00 | 4.19 | 1.26 |
| Poster rating self-enhancement | 1.00 | 7.00 | 3.90 | 1.24 |

Note. Pre-studies: N = 901. 449 (49.8%) male, 448 (49.7%) female, 3 (0.3%) non-binary, 1 (0.1%) trans male; Main study: N = 404. 202 (50%) male, 200 (49.5%) female, 2 (0.5%) non-binary.

Supplementary Table 2. Intercorrelations of main variables.

|  | Value: self-transcendent | Value: self-enhancement | Poster ratings self-transcendent |
| --- | --- | --- | --- |
|  | r | r | r |
| pre-studies |  |  |  |
| Value: self-enhancement | .08* |  |  |
| Poster ratings self-transcendent | .37** | .08* |  |
| Poster ratings self-enhancement | .29** | .19** | .68** |
| main study |  |  |  |
| Value: self-enhancement | .23** |  |  |
| Poster ratings self-transcendent | .42** | .18** |  |
| Poster ratings self-enhancement | .23** | .24** | .68** |

Note. Pre-studies: N = 901; main study: N = 404; * p < .05, ** p < .01

Supplementary Table 3. Intercorrelations of items used for poster-ratings.

|  | Item 2 | Item 3 | Item 4 |
| --- | --- | --- | --- |
|  | r | r | r |
| Item 1 | .883** | .825** | .800** |
| Item 2 |  | .866** | .815** |
| Item 3 |  |  | .820** |

Supplementary Table 4. Detailed regression results predicting self-transcendent poster ratings.

|  | poster ratings |  |  |  |  |
| --- | --- | --- | --- | --- | --- |
|  | self-transcendent |  |  |  |  |
| Value | b | SE | 95% CI | β | *p* |
| pre-studies |  |  |  |  |  |
| model 1 |  |  |  |  |  |
| intercept | 1.097 | 0.305 | (0.498; 1.696) |  |  |
| self-transcendent | 0.509 | 0.043 | (0.425; 0.593) | .370 | <.001 |
| self-enhancement | 0.067 | 0.046 | (-0.023; 0.156) | .045 | .146 |
| R^2 | .14 |  |  |  |  |
| model 2 |  |  |  |  |  |
| intercept | 0.639 | 0.234 | (0.179; 1.099) |  |  |
| self-transcendent | 0.263 | 0.034 | (0.197; 0.33) | .191 | <.001 |
| self-enhancement | -0.094 | 0.035 | (-0.163; -0.024) | -.064 | .009 |
| other poster-rating | 0.635 | 0.025 | (0.585; 0.684) | .635 | <.001 |
| R^2 | .50 |  |  |  |  |
| main study |  |  |  |  |  |
| model 1 |  |  |  |  |  |
| intercept | 0.562 | 0.427 | (-0.278; 1.401) |  |  |
| self-transcendent | 0.537 | 0.063 | (0.414; 0.661) | .396 | <.001 |
| self-enhancement | 0.140 | 0.072 | (-0.003; 0.282) | .089 | .054 |
| R^2 | .18 |  |  |  |  |
| model 2 |  |  |  |  |  |
| intercept | -0.153 | 0.327 | (-0.795; 0.489) |  |  |
| self-transcendent | 0.382 | 0.048 | (0.286; 0.477) | .282 | <.001 |
| self-enhancement | -0.049 | 0.056 | (-0.159; 0.061) | -.032 | .378 |
| other poster-rating | 0.627 | 0.036 | (0.555; 0.698) | .619 | <.001 |
| R^2 | .53 |  |  |  |  |

Supplementary Table 5. Detailed regression results predicting self-enhancement poster ratings.

|  | poster ratings |  |  |  |  |
| --- | --- | --- | --- | --- | --- |
|  | self-enhancement |  |  |  |  |
| Value | b | SE | 95% CI | β | *p* |
| pre-studies |  |  |  |  |  |
| model 1 |  |  |  |  |  |
| intercept | 0.722 | 0.310 | (0.113; 1.331) |  |  |
| self-transcendent | 0.387 | 0.043 | (0.301; 0.472) | .281 | <.001 |
| self-enhancement | 0.252 | 0.046 | (0.161; 0.343) | .171 | <.001 |
| R^2 | .12 |  |  |  |  |
| model 2 |  |  |  |  |  |
| intercept | 0.004 | 0.239 | (-0.465; 0.472) |  |  |
| self-transcendent | 0.053 | 0.036 | (-0.017; 0.124) | .039 | .135 |
| self-enhancement | 0.209 | 0.036 | (0.139; 0.278) | .142 | <.001 |
| other poster-rating | 0.655 | 0.026 | (0.604; 0.706) | .654 | <.001 |
| R^2 | .48 |  |  |  |  |
| main study |  |  |  |  |  |
| model 1 |  |  |  |  |  |
| intercept | 1.141 | 0.445 | (0.266; 2.015) |  |  |
| self-transcendent | 0.248 | 0.065 | (0.12; 0.377) | .186 | <.001 |
| self-enhancement | 0.302 | 0.075 | (0.153; 0.45) | .196 | <.001 |
| R^2 | .09 |  |  |  |  |
| model 2 |  |  |  |  |  |
| intercept | 0.759 | 0.338 | (0.094; 1.423) |  |  |
| self-transcendent | -0.117 | 0.054 | (-0.223; -0.011) | -.087 | .031 |
| self-enhancement | 0.207 | 0.057 | (0.094; 0.319) | .134 | <.001 |
| other poster-rating | 0.680 | 0.039 | (0.603; 0.758) | .689 | <.001 |
| R^2 | .48 |  |  |  |  |

Supplementary Material 1: Posters

| 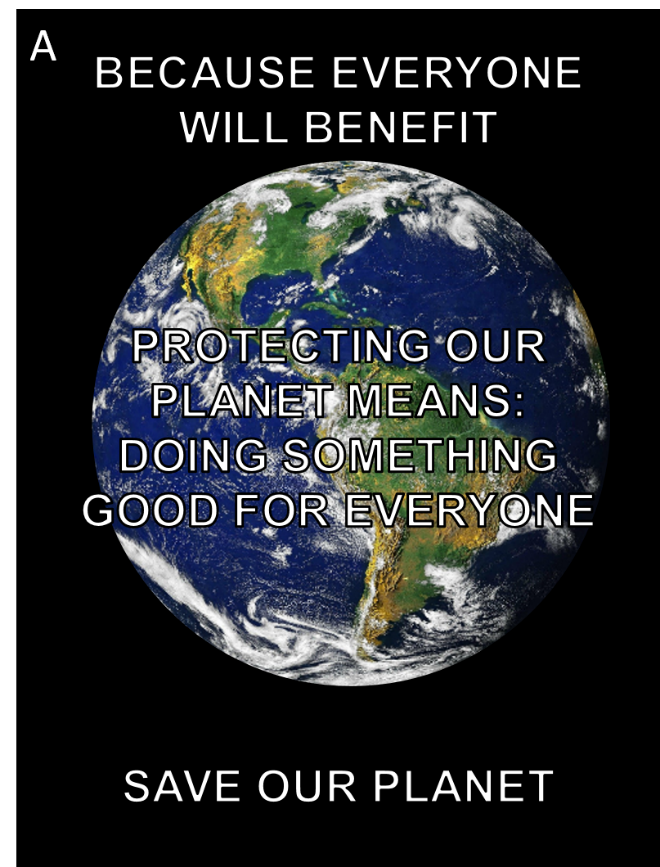 | 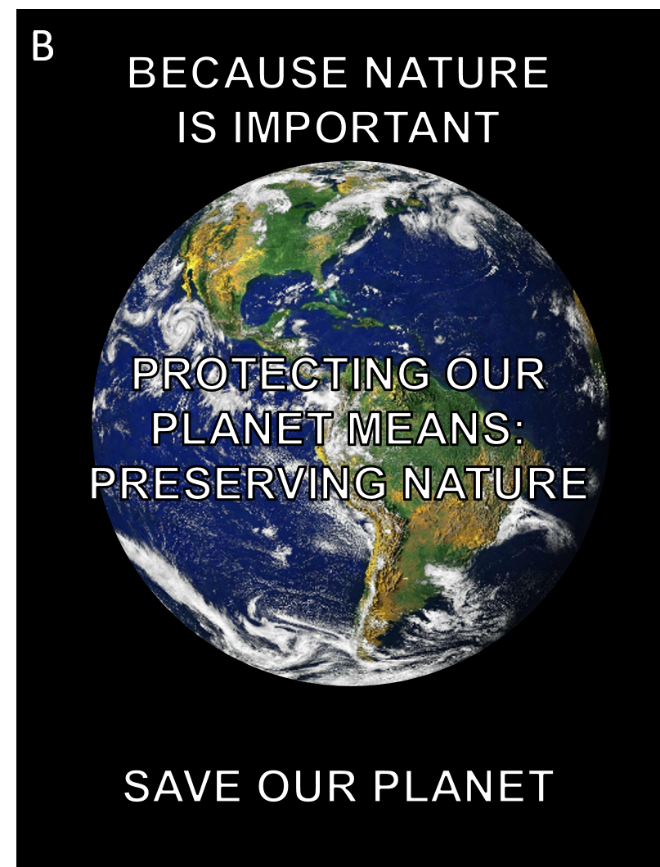 |
| --- | --- |
| 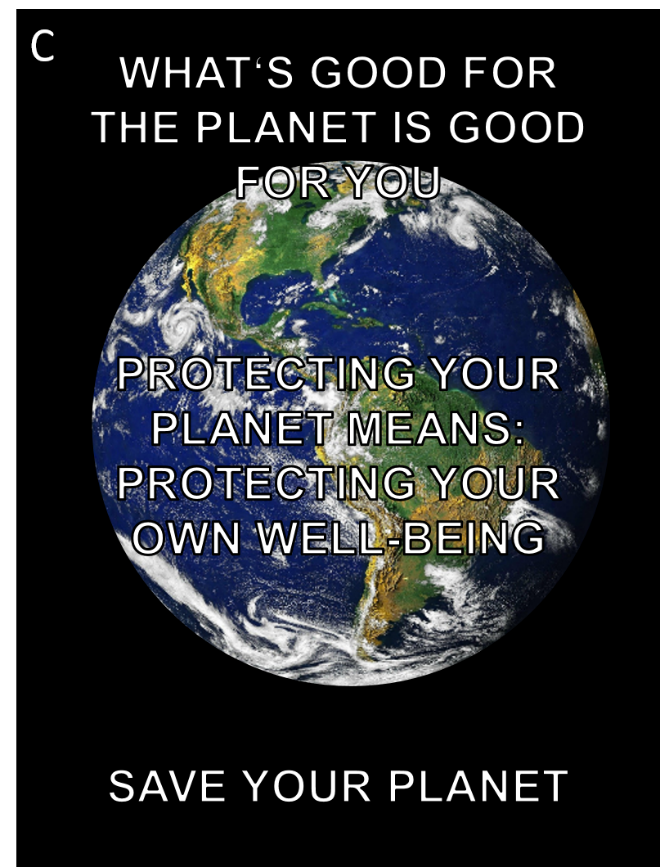 | 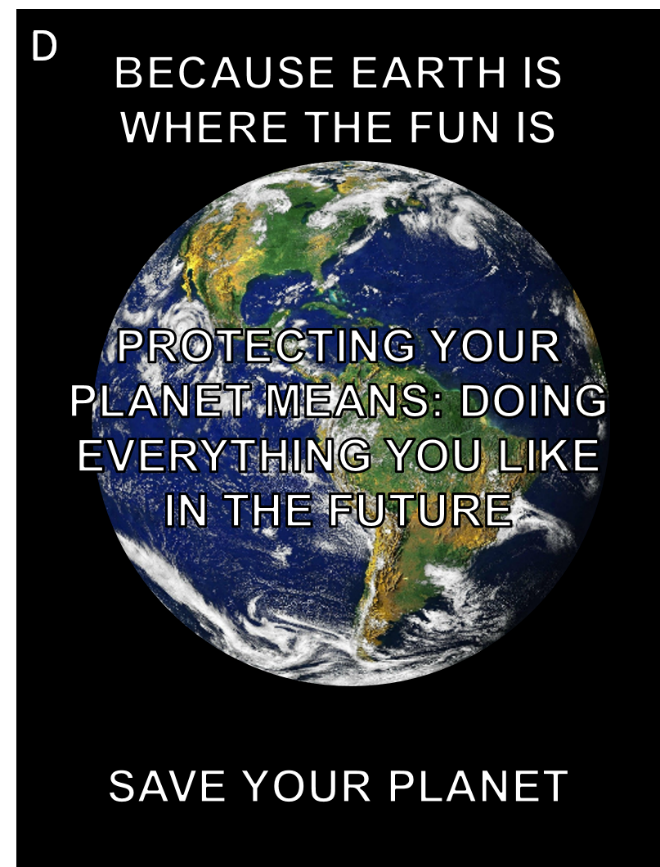 |

*Legend* A: Altruistic poster, B: Biospheric poster, C: Egoistic poster, D: Hedonic poster. Satellite image provided by WikiImages via pixabay (https://pixabay.com/photos/earth-globe-planet-world-space-11015/), under the CC0 License.
